# Benzoin in the Treatment of Chronic Dysentery

**Published:** 1856-11

**Authors:** 


*Benzoin in the Treatment of Chronic Dysentery.*

The compound tincture of Benzoin, in the dose of from fifteen to twenty minims, is strongly recommended by Mr. Wells, of Bristol, as a valuable remedy in chronic dysentery.—*London Lancet.*

